# Diurnal stability of cell-free DNA and cell-free RNA in human plasma samples

**DOI:** 10.1038/s41598-020-73350-3

**Published:** 2020-10-05

**Authors:** Josiah T. Wagner, Hyun Ji Kim, Katie C. Johnson-Camacho, Taylor Kelley, Laura F. Newell, Paul T. Spellman, Thuy T. M. Ngo

**Affiliations:** 1 Knight Cancer Institute, Cancer Early Detection Advanced Research Center (CEDAR), Oregon Health & Science University, Portland, OR 97201 USA; 2 Department of Biomedical Engineering, Oregon Health & Science University, Portland, OR 97239 USA; 3 Knight Cancer Institute, Oregon Health & Science University, Portland, OR 97201 USA; 4 Knight Cancer Institute, Hematology and Medical Oncology, Oregon Health & Science University, Portland, OR 97201 USA; 5 Department of Molecular and Medical Genetics, Oregon Health & Science University, Portland, OR 97239 USA; 6 Computational Biology Program, Oregon Health & Science University, Portland, OR 97239 USA

**Keywords:** Biomarkers, Medical research, Gene expression analysis, Genomic analysis, Isolation, separation and purification, Physiology

## Abstract

Many emerging technologies are reliant on circulating cell-free DNA (cfDNA) and cell-free RNA (cfRNA) applications in the clinic. However, the impact of diurnal cycles or daily meals on circulating analytes are poorly understood and may be confounding factors when developing diagnostic platforms. To begin addressing this knowledge gap, we obtained plasma from four healthy donors serially sampled five times during 12 h in a single day. For all samples, we measured concentrations of cfDNA and cfRNA using both bulk measurements and gene-specific digital droplet PCR. We found no significant variation attributed to blood draw number for the cfDNA or cfRNA. This indicated that natural diurnal cycles and meal consumption do not appear to significantly affect abundance of total cfDNA, total cfRNA, or our two selected cfRNA transcripts. Conversely, we observed significant variation between individual donors for cfDNA and one of the cfRNA transcripts. The results of this work suggest that it will be important to consider patient-specific baselines when designing reliable circulating cfDNA or cfRNA clinical assays.

## Introduction

Liquid-biopsy based diagnostic platforms are highly desired and increasingly accepted in clinical settings^1^. Although many diseases could potentially benefit from liquid-biopsy technology, the need for non-invasive platforms is especially apparent in the field of cancer screening and diagnostics because of potential risks involved with invasive needle biopsy procedures (e.g.^2,3^) and concerns of radiation exposure during imaging tests^4,5^. In addition, there is substantial interest in accurate screening methods that can be performed at frequent intervals to stratify patient cancer risk and detect potentially lethal cancers at early, treatable stages^6^. Plasma or serum-based platforms are of particular interest because the circulatory system interacts with the entire body and therefore provides a means to sample all organs. The sensitivity of current methods to reliably characterize nucleotide sequences and subcellular particles at single-event resolution has generated substantial interest in utilizing circulating cell-free DNA (cfDNA, e.g.^7–10^) and cell-free RNA (cfRNA, e.g.^11–15^) as clinically-relevant biomarkers. Human plasma contains both vesicular and extravesicular RNA and DNA; these different components may have distinctive contents with potential clinical relevance^16^. While promising, translation of cfDNA and cfRNA to the clinic has been slow, in part, because the natural temporal and interpersonal variation of the circulating analytes remains poorly understood. It is well-known that mammalian blood cell/tissue gene expression and physiology changes drastically during the daily diurnal cycle or following meals^17–20^. Recent evidence also suggests that some human bodily fluid-derived micro-RNAs (miRNAs) may follow a daily cycle of fluctuation^21,22^. Therefore, establishing the extent to which circulating cfDNA and cfRNA is affected by normal physiology is critical for the analytes to have successful clinical implementation.

To address the potential influence of daily cycles on circulating analytes, we characterized total cfDNA and cfRNA in plasma obtained from four healthy volunteers sampled multiple times across 2 days. We measured bulk cfDNA and cfRNA concentration using fluorometric or automated electrophoresis methods, as well as sequence-specific cfDNA and cfRNA copy number concentration using digital droplet PCR (ddPCR). The droplet-counting approach of ddPCR allows for absolute quantitation of nucleic acid templates and more sensitive resolution of fold-changes compared to conventional quantitative PCR^23–25^. Using this ddPCR approach, we targeted two single-copy genomic DNA regions as proxies for cfDNA abundance: one locus containing the gene *telomerase reverse transcriptase* (*TERT*) and one locus containing the gene *N-acetylglucosamine kinase* (*NAGK*); for cfRNA we targeted two commonly used genes used for mRNA normalization: *β-actin* (*ACTB*) and *glyceraldehyde-3-phosphate dehydrogenase* (*GAPDH*). Our results suggest that while cfDNA and cfRNA are overall stably expressed diurnally, several of the analytes demonstrate significant interpersonal or daily variation. Deeper characterization of these sources of variation will likely be required before the circulating analytes gain greater acceptance as clinically practical liquid-biopsy platforms.

## Methods

### Participant plasma sample collection

**Figure 1:**
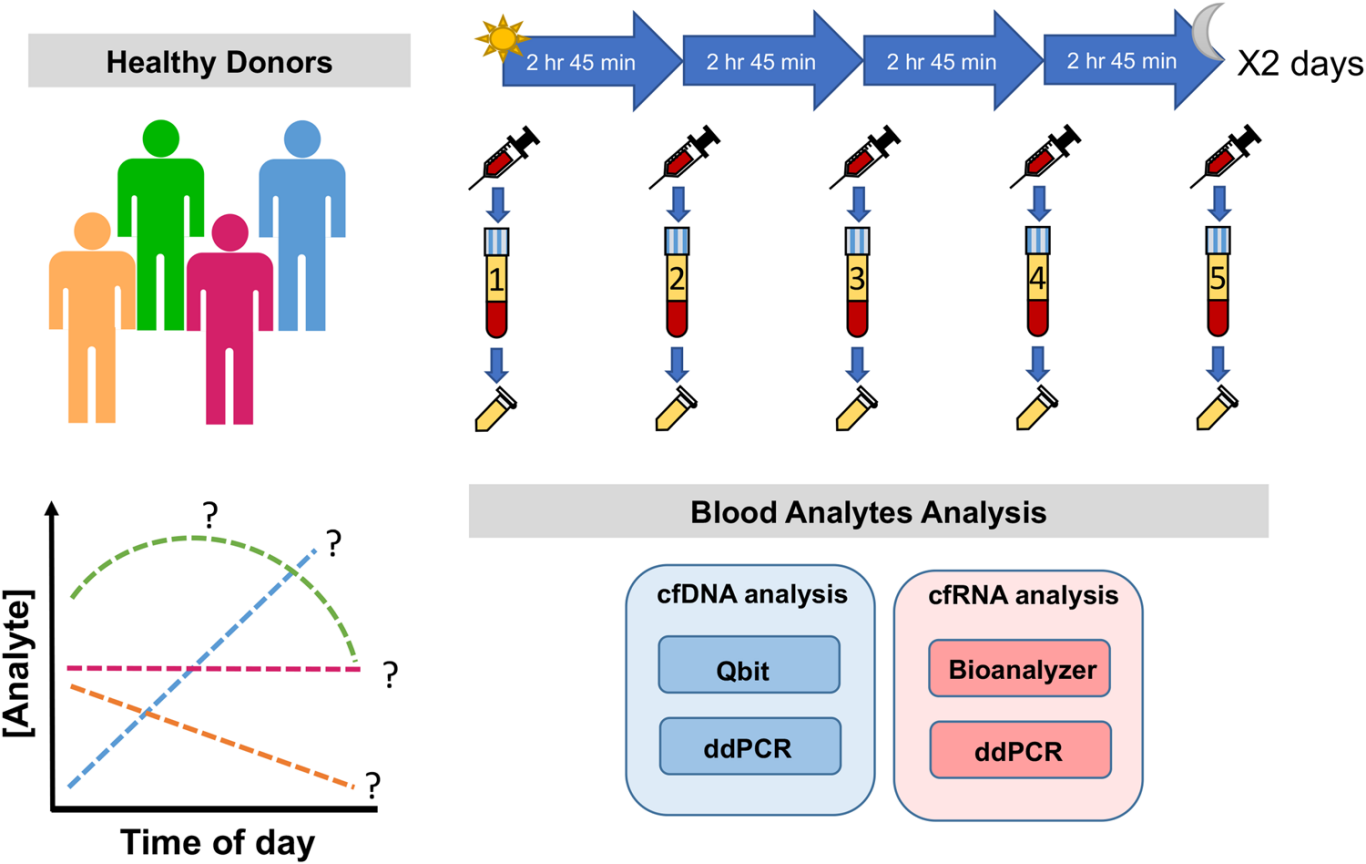
Schematic of the HD sampling procedure used to obtain plasma for analysis. Blood from four HDs were sampled five times per day over two days. The blood was processed into plasma and analyzed for changes in cfDNA or cfRNA.

All experimental protocols were reviewed and approved by the Oregon Health & Science University Institutional Review Board (protocol #8316). All methods were carried out in accordance with relevant guidelines and regulations. Informed consent was obtained from all volunteers, and volunteers were compensated for participating. Healthy donors (HDs) were consented for multiple blood draws over a 12-h period at the Oregon Health & Science University Oregon Clinical and Translational Research Institute (OCTRI) inpatient research clinic. HD age and sex distributions are given in Table 1. Each HD had 2 days, separated by 1 week, to provide five blood draws each day (Fig. 1). The HDs were advised to not engage in rigorous exercise 24 h prior to the blood draw dates. HDs were given access to recliner chairs and had the ability to walk around freely between blood draws. All individuals had an IV inserted for the multiple blood draws, but at times when the IV failed, ad hoc venipuncture was performed about 50% of the time for each participant. Blood was drawn from individuals every 2 h 45 min beginning at 8:30 am. Meals ordered from the hospital menu were consumed by individuals between draw 1–2, draw 2–3, and draw 4–5. To replicate a typical patient arriving in a clinical setting, diets were not restricted. Approximately 20 ml of blood was drawn into 10 ml EDTA tubes (Cat# 366643, BD Vacutainer) per time point. Blood was processed within 15 min of the draw and plasma was obtained as follows: EDTA tubes were centrifuged at 1000 × *g* for 10 min at room temperature, plasma was extracted down to ~ 500 μl from the buffy coat interface, plasma supernatant was centrifuged for a second time at 2500 × *g* for 10 min at room temperature, and the resulting plasma supernatant was extracted down to ~ 200 μl from the debris pellet interface. The final supernatant was distributed into 1 ml aliquots and immediately frozen at − 80 °C until analysis.

**Table 1:** Age and sex of the four healthy donors (HDs) that volunteered for this study.

Identifier	Age (years)	Sex
HD1	42.5	F
HD2	50	M
HD3	60.1	F
HD4	73.7	F

### Nucleic acid extractions

For each HD and time point, cfDNA and cfRNA was extracted separately (Fig. 1). CfDNA was extracted from 1 ml plasma using the QIAamp Circulating Nucleic Acid Kit (Qiagen #55114) according to manufacturer instructions and eluted into 20 μl buffer EB (10 mM Tris–Cl, pH 8.5). CfRNA was extracted from 1 ml plasma using the Plasma/Serum Circulating and Exosomal RNA Purification Kit (Norgen #42800) and eluted into 100 μl nuclease-free deionized water. To remove genomic DNA contamination, the cfRNA samples were treated with 2 MBU of Baseline-ZERO DNase in 1X Baseline-ZERO DNAse buffer (Lucigen #DB0715K) for 20 min at 37 °C, purified using the RNA Clean & Concentrator-5 kit (Zymo #R1013), and eluted in 14 μl nuclease-free water. For no plasma controls, 1 ml nuclease-free deionized water was used in place of plasma. These purified cfDNA and cfRNA samples were used for all subsequent nucleic acid analyses.

### Bulk quantitation of cfDNA and cfRNA

Purified cfDNA concentration was first determined using the Qubit dsDNA HS Assay Kit (quantification range 0.2–100 ng DNA, Thermo Fisher Scientific #Q32854) and Qubit 3 Fluorometer (Thermo Fisher Scientific). Purified cfRNA concentration was quantified using the Agilent RNA 6000 Pico Kit (quantification range 50–5000 pg/μL RNA in water, Agilent #5067–1513) and Agilent 2100 Bioanalyzer instrument (Agilent) within a window of 50–500 bp.

### First-strand cDNA synthesis of cfRNA

For first-strand cDNA synthesis of cfRNA, 10 μl reverse transcription reactions were prepared using 3 μl total cfRNA in 1X SuperScript IV VILO Mastermix (Thermo Fisher Scientific #11756050). cDNA synthesis reactions were incubated at 25 °C for 10 min, followed by 50 °C for 10 min, and were terminated with incubation at 85 °C for 5 min. The cDNA reactions were used as direct template for cDNA copy number quantification by ddPCR.

### Primers for ddPCR analysis

Primers and probes for cfDNA ddPCR analysis to target single-copy number genes *TERT* (F primer 5′–3′: CCTCACATAAATGCTACCAAACGA; R primer 5′–3′: TTCCAAGAAGGAGGCCATAGTC; Probe 5′–3′: AAGAAATGAACAGACCCATCCCCCAGG; fluorescent probe: HEX; quencher: ZEN/IBFQ) or *NAGK* (F primer 5′–3′: TGGGCAGACACATCGTAGCA; R primer 5′–3′: CACCTTCACTCCCACCTCAAC; Probe 5′–3′: TGTTGCCCGAGATTGACCCGGT; fluorescent probe: FAM; quencher: ZEN/IBFQ) and were purchased from IDT (Integrated DNA Technologies, Coralville, IA, USA). Primer and probe sequences for cfDNA were chosen using sequences reported by Devonshire et al.^26^ for these two genomic loci. To quantify cDNA copy number, gene expression ddPCR assays for *ACTB* (assay dHsaCPE5190199; fluorescent probe: FAM) and *GAPDH* (assay dHsaCPE5031597; fluorescent probe: HEX) were purchased from Bio-Rad and contained both probes and primers premixed at 10X concentration.

### ddPCR of cfDNA and cDNA samples

To measure cfDNA copy number by ddPCR, 22 μl ddPCR reaction mixtures were prepared using 2.2 μl purified cfDNA in 1X ddPCR Supermix for Probes (No dUTP) (Bio-Rad #1863024) and with final primer/probe concentrations of 0.9 µM/0.25 µM or 0.2 µM/0.1 µM for *TERT* and *NAGK*, respectively. Each cfDNA ddPCR reaction was multiplexed for both *TERT* and *NAGK*. To measure cDNA copy number by ddPCR, 22 μl ddPCR reaction mixtures were prepared using 1.5 μl undiluted cDNA template in 1X ddPCR Supermix for Probes (No dUTP) (Bio-Rad #1863024), 1X *ACTB* gene expression ddPCR assay mix, and 1X *GAPDH* gene expression ddPCR assay mix. For no template controls (NTCs), 1 μl nuclease-free water was used instead of cfDNA or cDNA template. Reactions were performed in semi-skirted 96-well plates (Eppendorpf #951020362). Plates for droplet generation were heat-sealed with pierceable foil (Bio-Rad #1814040), vortexed briefly, then spun down using a tabletop plate spinner. Droplet generation was performed using a QX200 AutoDG Droplet Digital PCR System (Bio-Rad) with Automated Droplet Generation Oil for Probes (Bio-Rad #1864110) and DG32 Automated Droplet Generator Cartridges (Bio-Rad #1864108). Droplets were deposited into a clean 96-well plate held in a pre-chilled cold block to prevent evaporation. Plates were then heat-sealed with pierceable foil and PCR was performed in a Bio-Rad C1000 thermocycler using the following temperature conditions: 95 °C for 10 min, 40 cycles of 94 °C for 30 s followed by 60 °C for 1 min, 98 °C for 10 min, and then cooling to 4 °C until droplets were read. Droplets were counted using the QX200 Droplet Reader (Bio-Rad) using manufacturer’s instructions. Positive and negative droplets were subsequently analyzed using QuantaSoft Analysis Pro (v1.0.596, Bio-Rad, Hercules, California, USA).

### Statistics and plots

Graphs of cfDNA and cfRNA measured over time were prepared using R (v.3.6.1) and Rstudio (v.1.2.5019). Permutation tests for each analyte were performed in Rstudio using the “coin” R package and default parameters^27,28^. For significant permutation tests, post-hoc pairwise permutation tests were performed using the R package “rcompanion” (v. 2.3.2, https://rcompanion.org) with the “fdr” *P* value adjustment method. To test for a significant difference between draws performed on day 1 versus day 2, a permutation test of symmetry was performed on the draws paired by day. To test for a significant difference between draws or between individuals, the values between day 1 and day 2 for each draw were averaged and one-way permutation tests of independence were performed using either draws or individuals as factors. Summary statistics, correlation plots, Spearman nonparametric correlation coefficient, and two-tailed correlation *P* value analysis were prepared using GraphPad Prism (v8.3.0, GraphPad Software, San Diego, CA, USA). For all statistical tests, *P* values were determined to be significant at a threshold of *P* ≤ 0.05.

## Results and discussion

Over the past decade, it has become increasingly clear that detecting unpredictable disease via blood biopsy, especially at the earliest stages, will require intricate understanding of naturally occurring circulating biomarker variation. Workflow standardization for liquid-biopsy based analytes is the first step towards identifying and minimizing sources of non-biologically relevant variation^29–31^. However, confounding factors related to meals, time of day, and the intrinsic interpersonal variation could critically affect analyte abundance, normalization, and multi-omic integration. To begin addressing these concerns, here we provide the first descriptions of diurnal cfDNA and cfRNA derived from the same cohort.

### Differences in plasma cfDNA abundance across donors is attributed to interpersonal variation

**Figure 2:**
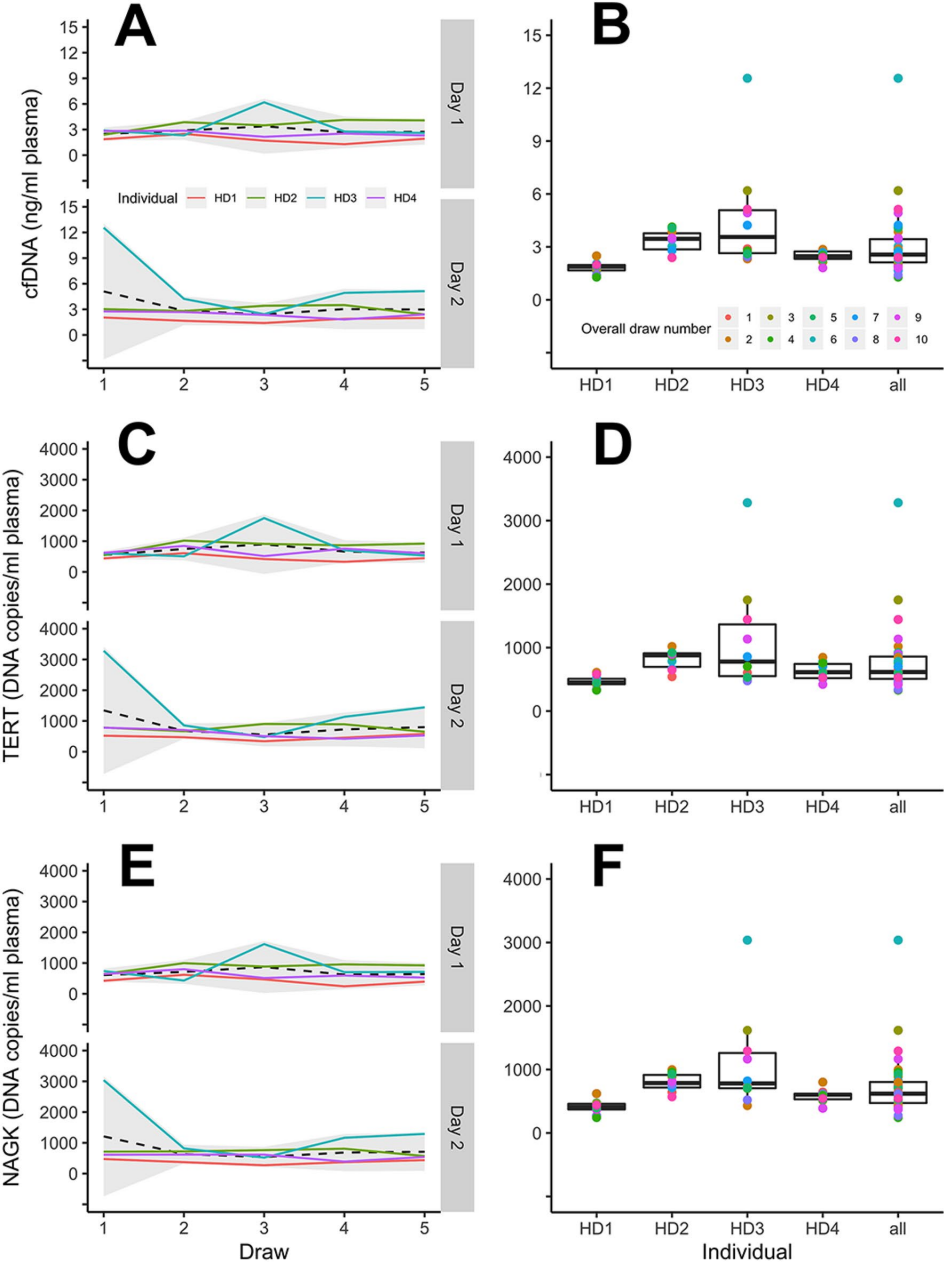
Abundance of plasma-derived cfDNA across the five sampled time points. CfDNA abundance was measured by Qubit (**A**, **B**) and by ddPCR with TERT (**C**, **D**) or NAGK probes (**E**, **F**). For left panels, dashed lines represent the average of the four individuals and the shaded area corresponds to 95% confidence intervals. For right panels, the individual data points overlaying boxplots are color coded sequentially starting from the first blood draw of day 1.

First, we characterized total donor plasma cfDNA abundance using Qubit as well as total genome copy numbers via locus-specific ddPCR assays. Following cfDNA extractions of plasma from the four HDs, we observed overall averages of 3.05 ng cfDNA / ml plasma when measured by Qubit (SD = 1.2 ng cfDNA / ml plasma, Fig. 2A,B, Supplemental table S1). When cfDNA was measured by ddPCR, we observed 758.6 copies/ml plasma (SD = 286.2) and 723.5 copies/ml plasma (SD = 299.3) using TERT and NAGK probes, respectively (Figs. 2C–F). The ddPCR workflow for cfDNA yielded an average of 14,658 accepted droplets (range = 10,426–18,130; SD = 1366) per ddPCR reaction well (Supplemental Figure S1). No plasma controls and NTCs for TERT/NAGK ranged from 0–9.3 copies/ml (Supplemental Figure S2). We observed a strong correlation between the two cfDNA extraction replicates (Spearman rs = 0.73, *P* < 0.0001, Figure S3A) and between the two analysis methods (Spearman rs = 0.97, *P* < 0.0001, Figure S3B). We found no significant difference in cfDNA abundance between the five draws when measured by either Qubit or ddPCR (Table 2, Supplemental tables S1–S3). This finding contrasts with a recent report by Madsen et al.^32^ which found a decrease in plasma cfDNA concentration at their final draw when five draws were performed three hours apart. However, our second centrifugation step was performed at 2500 × *g*, rather than 13,000 × *g* as done by Madsen et al.^32^, and therefore the composition of cell-free plasma may not be directly comparable. We observed no significant difference between the two draw days (Table 2), although we did observe a significant source of variation attributed to the individuals (*P* < 0.05, Table 2, Supplemental Table S1). For example, the cfDNA level of individual HD1 was consistently lower than the overall average (Fig. 2A, B, Supplemental Tables S1–S3). Post-hoc pairwise permutation tests also identified individuals with significantly different cfDNA levels (Supplemental Tables S1–S3). Plasma cfDNA was previously described by Zhong et al.^33^ to fluctuate 1.9–67.9 fold in healthy, nonpregnant individuals when sampled across 12-h or longer time points. Similar to Zhong et al., we observed inconsistent cfDNA fluctuation across time in the HDs and attribute the primary source of plasma cfDNA abundance differences to be from interpersonal variation.

**Table 2:** Summary of the permutation tests comparing the two draw days, the five draws across the day, or individuals to determine significant sources of variation.

Analyte	Analysis method	Day^a^	Draw number^b^	Individual^b^
cfDNA	Qubit	0.30 (ns)	0.38 (ns)	0.013 (*)
	TERT	0.61 (ns)	0.38 (ns)	0.021 (*)
	NAGK	0.35 (ns)	0.38 (ns)	0.020 (*)
cfRNA	Bioanalyzer	0.75 (ns)	0.64 (ns)	0.82 (ns)
	ACTB ddPCR	0.96 (ns)	0.80 (ns)	0.20 (ns)
	GAPDH ddPCR	0.29 (ns)	0.53 (ns)	0.016 (*)

^a^Permutation test of symmetry. *P* values are followed by *P* value summaries in parenthesis. *Ns* not significant.

^b^One-way permutation test of independence. *P* values are followed by *P* value summaries in parenthesis. *P < 0.05; **P < 0.01; ****P < 0.0001; *ns* not significant.

### GAPDH *counts, but not* ACTB *counts or total cfRNA, varied significantly by donor*

**Figure 3:**
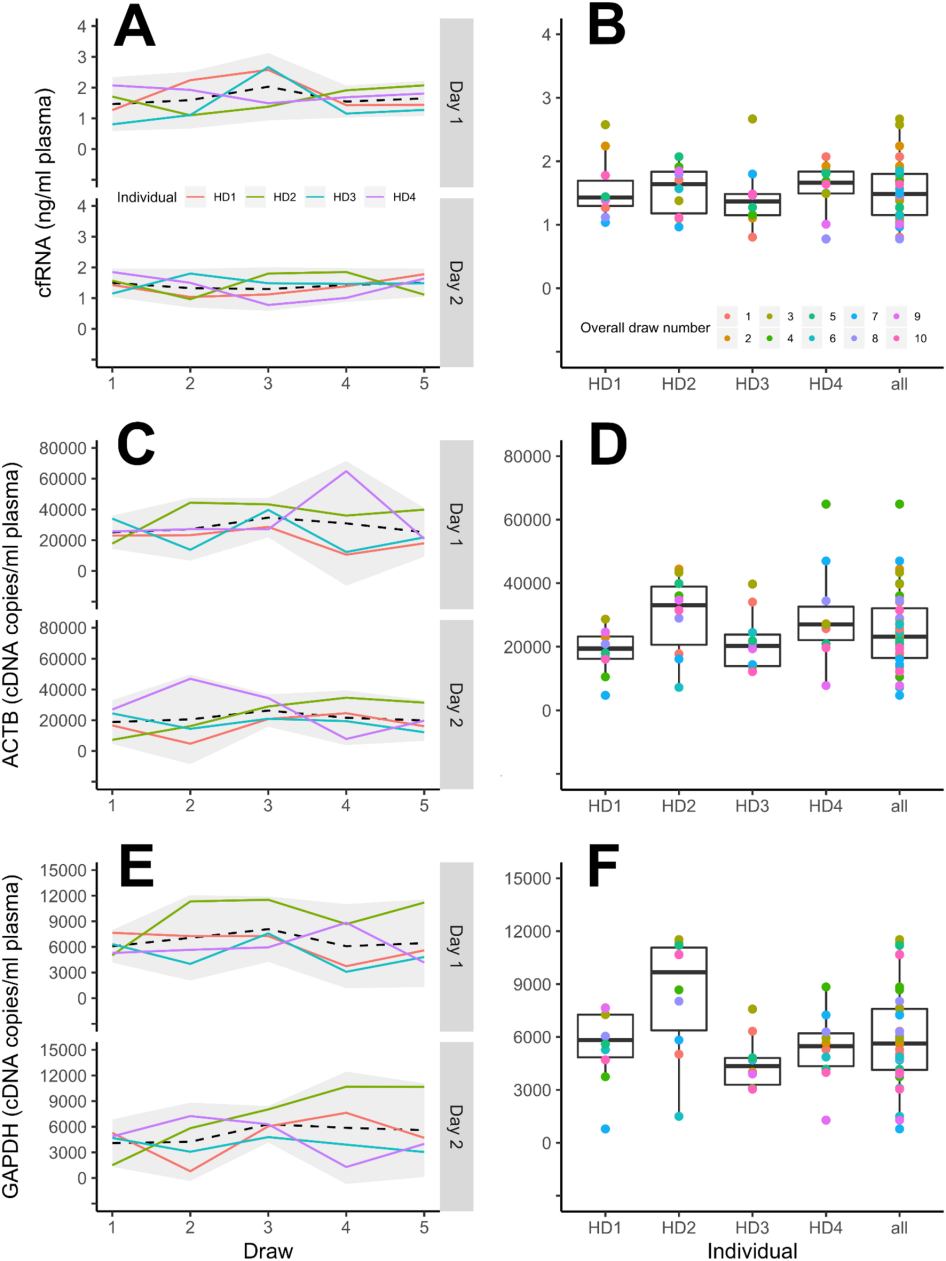
Abundance of plasma-derived cfRNA across the five sampled time points. CfRNA abundance was measured by Bioanalyzer (**A**, **B**) and by ddPCR with probes targetting *ACTB* (**C**, **D**) or *GAPDH* (**E**, **F**). For left panels, dashed lines represent the average of the four individuals and the shaded area corresponds to 95% confidence intervals. For right panels, the individual data points overlaying boxplots are color coded sequentially starting from the first blood draw of day 1.

Next, we measured total plasma cfRNA abundance by Bioanalyzer and mRNA-specific abundance characterization using ddPCR. Across the four HDs, we observed an overall average of 3.05 ng/ml plasma cfRNA when measured by Bioanalyzer (SD = 1.2 ng/ml plasma, Fig. 3A,B, Supplemental table S4). We did not find significant cfRNA variation by day, draw, or individual when total cfRNA abundance was measured using this method (Table 2). When cfRNA was measured by ddPCR, we observed 25022 copies/ml plasma (SD = 5932) and 5983 copies/ml plasma (SD = 1703) using *ACTB* and *GAPDH* probes, respectively (Fig. 3C–F). The ddPCR workflow for cfRNA yielded an average of 16,216 droplets per well (range = 11,912–19,618; SD = 1554) for *ACTB* and *GAPDH* cDNA templates (Supplemental Figure S2). No plasma controls and NTCs for ACTB/GAPDH ranged from 0 to 4.3 copies/ml (Supplemental Figure S5). Similar to our bulk measurement of cfRNA by Bioanalyzer, we did not observe significant variation due to day of draw or draw number for either *ACTB* or *GAPDH* (Fig. 3C–F; Table 2, Supplemental Tables S5 and S6). However, we observed significant variation attributed to the individuals for *GAPDH* counts (*P* < 0.05, Table 2). Post-hoc pairwise permutation tests for *GAPDH* counts did not reveal specific significant differences between individuals (Supplemental Tables S6).

*ACTB* and *GAPDH* are commonly used mRNA normalization genes for liquid biopsy applications^34,35^ despite accumulating evidence suggesting their expression can be highly variable and require situation-specific considerations^36–38^. Currently, there are few reports describing the long-term stability of plasma mRNA expression in individuals. Recent work by Max et al.^31^ found no significant variation miRNA plasma/serum profiles following meals and also described interpersonal differences in miRNA abundance that could be stable for up to a year. While we similarly did not find an effect of meals on bulk cfRNA abundance or *ACTB*/*GAPDH* expression, we found significant variation attributed to individual donors for *GAPDH* transcripts. Like our finding with plasma cfDNA abundance, our results suggest that at least some plasma-derived cfRNA transcripts may have baseline expression levels that are specific to the donor; therefore, a thorough understanding of cfRNA normalization transcripts across time and between individuals may be necessary for future cfRNA diagnostic applications.

## Conclusions

Liquid-biopsy technology for patient risk-stratification, diagnosis, or disease progression monitoring will likely require biomarker thresholds tailored specifically for each patient. In our pilot cohort, we observed significant interpersonal variation for each of the two analytes examined. Remarkably, distinct baseline levels of cfDNA and cfRNA of each individual were persistent through the draws over time. Future multi-omic studies such as the work presented here, but with larger and more inclusive cohorts, will be essential for determining the full extent of interpersonal and sample collection variation that may be present across populations.

## Supplementary information

Supplementary Information.
