# Supplementary material for: Diurnal stability of cell-free DNA and cell-free RNA in human plasma samples
Source: Sci Rep. 2020 Oct 5;10:16456. doi: 10.1038/s41598-020-73350-3 (PMC7536441; doi:10.1038/s41598-020-73350-3)
Supplement: Supplementary file 1 — Supplementary Information. [file 41598_2020_73350_MOESM1_ESM.docx]

**Supporting Information**

**Diurnal stability of cell-free DNA and cell-free RNA in human plasma samples**

Josiah T. Wagner^1*^, Hyun Ji Kim^1,2^, Katie C. Johnson-Camacho^1^, Taylor Kelley^3^, Laura F. Newell^4^, Paul Spellman^1,5,6^, Thuy T. M. Ngo^1,2,5*^

^1^Knight Cancer Institute Cancer Early Detection Advanced Research Center (CEDAR), Oregon Health & Science University, Portland, OR 97201, USA.

^2^Department of Biomedical Engineering, Oregon Health & Science University, Portland, OR 97201, USA.

^3^Knight Cancer Institute Precision Oncology, Oregon Health & Science University, Portland, OR 97201, USA.

^4^Knight Cancer Institute Hematology and Medical Oncology, Oregon Health & Science University, Portland, OR 97201, USA.

^5^Department of Molecular and Medical Genetics, Oregon Health & Science University, Portland, OR 97201, USA.

^6^Computational Biology Program, Oregon Health & Science University, Portland, OR 97201, USA.

*Correspondence to:

Josiah T. Wagner
[wagnejos@ohsu.edu](mailto:wagnejos@ohsu.edu)

Thuy T.M. Ngo
[ngth@ohsu.edu](mailto:ngth@ohsu.edu)


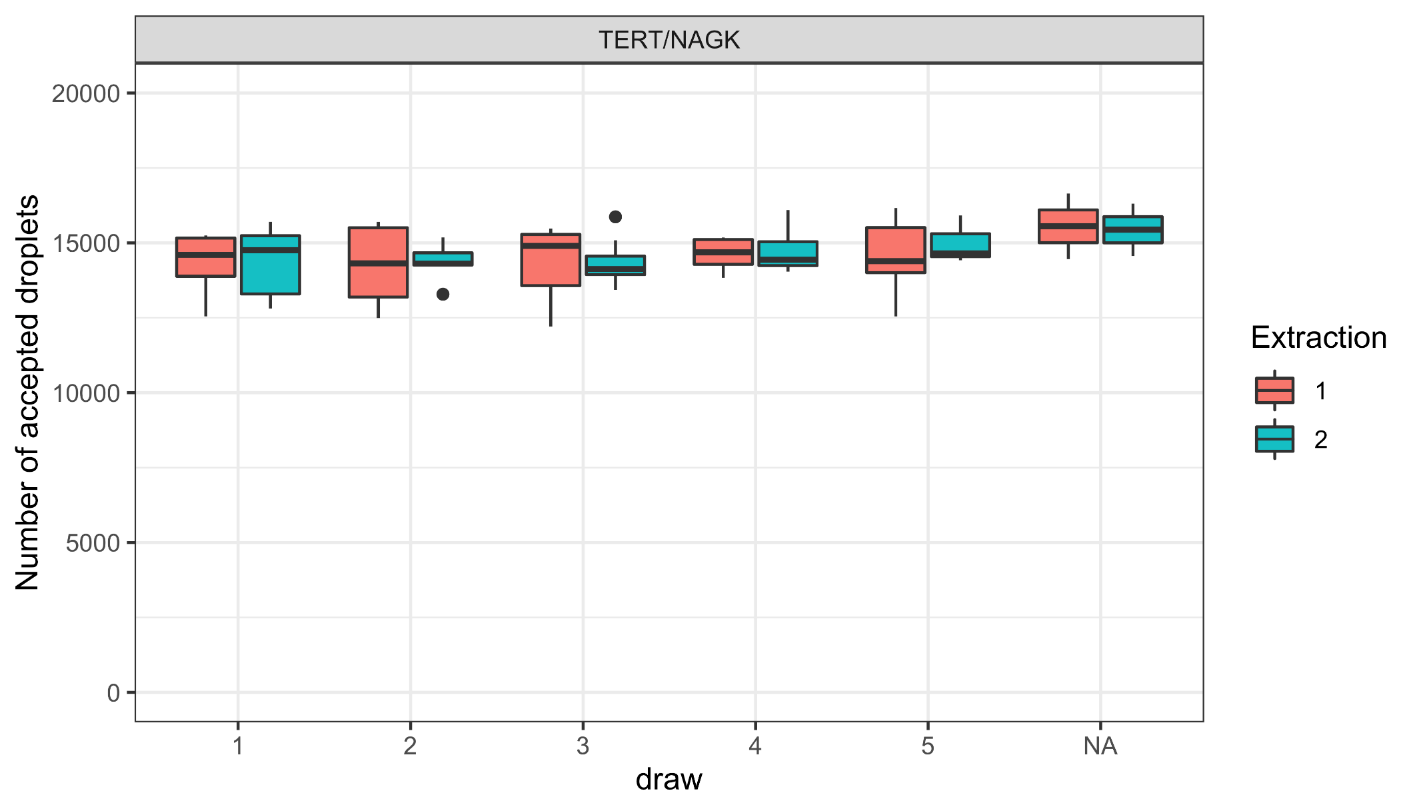


**Supplemental Figure S1. Total number of droplets accepted by the QX200 ddPCR droplet reader for nucleic acid quantitation.** Number of droplets for each draw are shown for cfDNA cfRNA ddPCR analysis. Technical replicates for each sample were averaged.


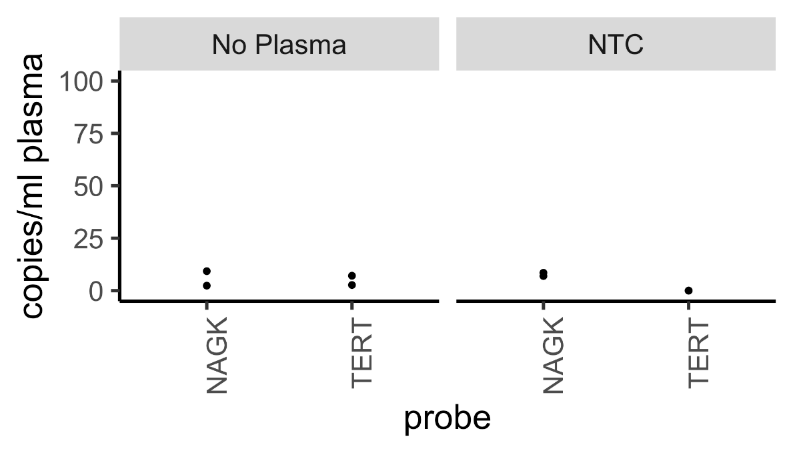


**Supplemental Figure S2. Negative controls for ddPCR measurement of cfDNA.** Data points are from the two independent cfDNA extractions performed in this work. Negative controls were measured using ddPCR at the same time as the plasma samples.

**Supplemental Figure S3.** Nonparametric Spearman correlation coefficients (rs) calculated between cfDNA extraction 1 and extraction 2 using Qubit measurements (A) and between Qubit and ddPCR (TERT and NAGK averaged) measurements of cfDNA (B). The correlations for both comparisons were statistically significant.


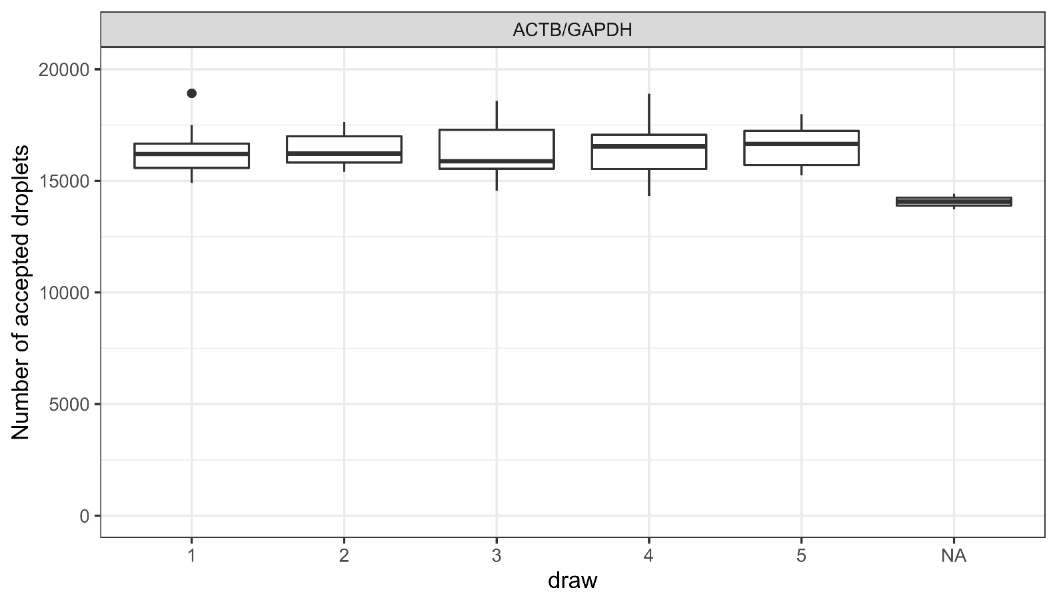


**Supplemental Figure S4. Total number of droplets accepted by the QX200 ddPCR droplet reader for nucleic acid quantitation.** Number of droplets for each draw are shown for cfDNA cfRNA ddPCR analysis. Technical replicates for each sample were averaged.


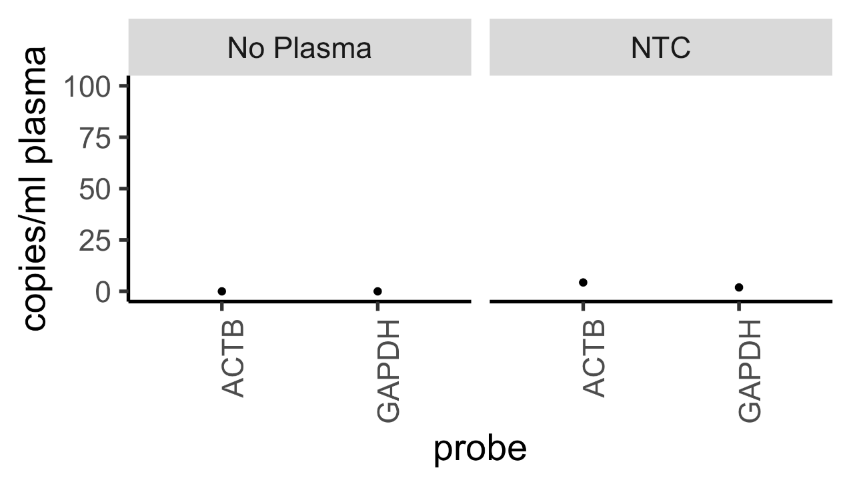


**Supplemental Figure S5. Negative controls for ddPCR measurement of cDNA derived from cfRNA.** Negative controls were measured using ddPCR at the same time as the plasma samples.

| **Supplemental Table S1.** Total plasma cfDNA concentration summaries and statistics as measured by Qubit. | | | | | | | | |
| --- | --- | --- | --- | --- | --- | --- | --- | --- |
|  | | **HD1** | **HD2** | | **HD3** | **HD4** | | **Overall** |
| **Number of values** | | 10 | 10 | | 10 | 10 | | 4 |
|  | |  |  | |  |  | |  |
| **Minimum^a^** | | 1.28 | 2.39 | | 2.32 | 1.81 | | 1.82 |
| **Median^a^** | | 1.88 | 3.46 | | 3.56 | 2.47 | | 2.89 |
| **Maximum^a^** | | 2.49 | 4.13 | | 12.56 | 2.86 | | 4.61 |
| **Mean^a^** | | 1.82 | 3.31 | | 4.61 | 2.47 | | 3.05 |
| **Std. Deviation^a^** | | 0.34 | 0.64 | | 3.10 | 0.33 | | 1.20 |
| **Lower 95% CI of mean^a^** | | 1.58 | 2.85 | | 2.39 | 2.23 | | 1.14 |
| **Upper 95% CI of mean^a^** | | 2.07 | 3.76 | | 6.82 | 2.71 | | 4.96 |
|  | |  |  | |  |  | |  |
| **Post-hoc pairwise comparison** | **P-value** | | | **Adjusted P** | | | **Summary**^b^ | |
| HD1 - HD2 | 0.005 | | | 0.030 | | | * | |
| HD1 - HD3 | 0.020 | | | 0.030 | | | * | |
| HD1 - HD4 | 0.016 | | | 0.030 | | | * | |
| HD2 - HD3 | 0.142 | | | 0.142 | | | ns | |
| HD2 - HD4 | 0.016 | | | 0.030 | | | * | |
| HD3 - HD4 | 0.040 | | | 0.049 | | | * | |

^a^ng per ml plasma

^b^ *, P < 0.05; ns, not significant.

| **Supplemental Table S2.** Plasma *TERT* concentration summaries and statistics as measured by ddPCR. | | | | | |
| --- | --- | --- | --- | --- | --- |
|  | **HD1** | **HD2** | **HD3** | **HD4** | **Overall** |
| **Number of values** | 10 | 10 | 10 | 10 | 4 |
|  |  |  |  |  |  |
| **Minimum^a^** | 329.5 | 541.8 | 476.9 | 420.5 | 461.2 |
| **Median^a^** | 452.2 | 878.9 | 779.8 | 612.5 | 722 |
| **Maximum^a^** | 610.3 | 1018 | 3282 | 846.4 | 1129 |
| **Mean^a^** | 461.2 | 815.5 | 1129 | 628.5 | 758.6 |
| **Std. Deviation^a^** | 90.9 | 150.3 | 870.2 | 139.2 | 286.2 |
| **Lower 95% CI of mean^a^** | 396.2 | 708 | 506.5 | 528.9 | 303.1 |
| **Upper 95% CI of mean^a^** | 526.3 | 923 | 1752 | 728.1 | 1214 |

| **Post-hoc pairwise comparison** | **P-value** | **Adjusted P** | **Summary**^b^ |
| --- | --- | --- | --- |
| HD1 - HD2 | 0.006 | 0.035 | * |
| HD1 - HD3 | 0.027 | 0.048 | * |
| HD1 - HD4 | 0.032 | 0.048 | * |
| HD2 - HD3 | 0.176 | 0.176 | ns |
| HD2 - HD4 | 0.032 | 0.048 | * |
| HD3 - HD4 | 0.060 | 0.073 | ns |

^a^copies per ml plasma

^b^ *, P < 0.05; ns, not significant.

| **Supplemental Table S3.** Plasma *NAGK* concentration summaries and statistics as measured by ddPCR. | | | | | |
| --- | --- | --- | --- | --- | --- |
|  | **HD1** | **HD2** | **HD3** | **HD4** | **Overall** |
| **Number of values** | 10 | 10 | 10 | 10 | 4 |
|  |  |  |  |  |  |
| **Minimum^a^** | 242.1 | 569.7 | 430.3 | 389.7 | 407 |
| **Median^a^** | 409.1 | 786.6 | 779.5 | 603.3 | 692 |
| **Maximum^a^** | 619.4 | 997.1 | 3037 | 800.5 | 1103 |
| **Mean^a^** | 407 | 798.3 | 1103 | 585.6 | 723.5 |
| **Std. Deviation^a^** | 106.6 | 141.4 | 771.9 | 106.2 | 299.3 |
| **Lower 95% CI of mean^a^** | 330.7 | 697.1 | 551 | 509.6 | 247.2 |
| **Upper 95% CI of mean^a^** | 483.2 | 899.5 | 1655 | 661.6 | 1200 |

| **Post-hoc pairwise comparison** | **P-value** | **Adjusted P** | **Summary**^b^ |
| --- | --- | --- | --- |
| HD1 - HD2 | 0.005 | 0.029 | * |
| HD1 - HD3 | 0.023 | 0.035 | * |
| HD1 - HD4 | 0.019 | 0.035 | * |
| HD2 - HD3 | 0.177 | 0.177 | ns |
| HD2 - HD4 | 0.015 | 0.035 | * |
| HD3 - HD4 | 0.051 | 0.061 | ns |

^a^copies per ml plasma

^b^ *, P < 0.05; ns, not significant.

| **Supplemental Table S4.** Total plasma cfRNA concentration summaries and statistics as measured by Bioanalyzer. | | | | | |
| --- | --- | --- | --- | --- | --- |
|  | **HD1** | **HD2** | **HD3** | **HD4** | **Overall** |
| **Number of values** | 10 | 10 | 10 | 10 | 4 |
|  |  |  |  |  |  |
| **Minimum^a^** | 1.036 | 0.966 | 0.805 | 0.777 | 1.439 |
| **Median^a^** | 1.428 | 1.638 | 1.369 | 1.663 | 1.558 |
| **Maximum^a^** | 2.576 | 2.072 | 2.667 | 2.072 | 1.575 |
| **Mean^a^** | 1.57 | 1.546 | 1.439 | 1.575 | 1.533 |
| **Std. Deviation^a^** | 0.492 | 0.3864 | 0.511 | 0.406 | 0.0636 |
| **Lower 95% CI of mean^a^** | 1.218 | 1.27 | 1.073 | 1.284 | 1.431 |
| **Upper 95% CI of mean^a^** | 1.922 | 1.823 | 1.804 | 1.866 | 1.634 |

^a^ng per ml plasma

| **Supplemental Table S5.** Plasma *ACTB* cDNA concentration summaries and statistics as measured by ddPCR. | | | | | |
| --- | --- | --- | --- | --- | --- |
|  | **HD1** | **HD2** | **HD3** | **HD4** | **Overall** |
| **Number of values** | 10 | 10 | 10 | 10 | 4 |
|  |  |  |  |  |  |
| **Minimum^a^** | 4,739 | 7,230 | 12,188 | 7,812 | 18,632 |
| **Median^a^** | 19,413 | 33,055 | 20,199 | 27,020 | 25,652 |
| **Maximum^a^** | 28,658 | 44,420 | 39,692 | 64,888 | 30,153 |
| **Mean^a^** | 18,632 | 29,989 | 21,314 | 30,153 | 25,022 |
| **Std. Deviation^a^** | 7,069 | 12,483 | 9,323 | 15,813 | 5,932 |
| **Lower 95% CI of mean^a^** | 13,575 | 21,059 | 14,645 | 18,841 | 15,582 |
| **Upper 95% CI of mean^a^** | 23,689 | 38,918 | 27,983 | 41,466 | 34,462 |

^a^copies per ml plasma

| **Supplemental Table S6.** Plasma *GAPDH* cDNA concentration summaries and statistics as measured by ddPCR. | | | | | |
| --- | --- | --- | --- | --- | --- |
|  | **HD1** | **HD2** | **HD3** | **HD4** | **Overall** |
| **Number of values** | 10 | 10 | 10 | 10 | 4 |
|  |  |  |  |  |  |
| **Minimum^a^** | 786 | 1,498 | 3,040 | 1,284 | 4,532 |
| **Median^a^** | 5,821 | 9,666 | 4,353 | 5,479 | 5,478 |
| **Maximum^a^** | 7,656 | 11,525 | 7,582 | 8,839 | 8,444 |
| **Mean^a^** | 5,597 | 8,444 | 4,532 | 5,359 | 5,983 |
| **Std. Deviation^a^** | 2,151 | 3,372 | 1,484 | 2,031 | 1,703 |
| **Lower 95% CI of mean^a^** | 4,059 | 6,032 | 3,471 | 3,907 | 3,273 |
| **Upper 95% CI of mean^a^** | 7,136 | 10,856 | 5,594 | 6,812 | 8,693 |

| **Post-hoc pairwise comparison** | **P-value** | **Adjusted P** | **Summary**^b^ |
| --- | --- | --- | --- |
| HD1 - HD2 | 0.084 | 0.169 | ns |
| HD1 - HD3 | 0.169 | 0.254 | ns |
| HD1 - HD4 | 0.695 | 0.695 | ns |
| HD2 - HD3 | 0.039 | 0.169 | ns |
| HD2 - HD4 | 0.067 | 0.169 | ns |
| HD3 - HD4 | 0.245 | 0.294 | ns |

^a^copies per ml plasma

^b^ns, not significant.
